# Transformation Scoring System (TSS): A new assessment index for clinical transformation of follicular lymphoma

**DOI:** 10.1002/cam4.3501

**Published:** 2020-10-06

**Authors:** Takafumi Shichijo, Dai Maruyama, Nobuhiko Yamauchi, Akiko Miyagi Maeshima, Masato Sugano, Sayako Yuda, Kinuko Tajima, Hiroaki Kurihara, Kaoru Shimada, Tomotaka Suzuki, Kosuke Toyoda, Shinichi Makita, Suguru Fukuhara, Wataru Munakata, Tatsuya Suzuki, Yukio Kobayashi, Hirokazu Taniguchi, Yosuke Minami, Koji Izutsu, Kensei Tobinai

**Affiliations:** ^1^ Department of Hematology National Cancer Center Hospital Tokyo Japan; ^2^ Department of Hematology National Cancer Center Hospital East Kashiwa Japan; ^3^ Department of Pathology and Clinical Laboratories National Cancer Center Hospital Tokyo Japan; ^4^ Department of Pathology and Clinical Laboratories National Cancer Center Hospital East Kashiwa Japan; ^5^ Department of Diagnostic Radiology National Cancer Center Hospital Tokyo Japan; ^6^ Department of Diagnostic Radiology National Cancer Center Hospital East Kashiwa Japan

**Keywords:** clinical transformation, diffuse large B‐cell lymphoma, follicular lymphoma, histologic transformation, scoring system

## Abstract

Although histologic analysis is the gold standard for diagnosing follicular lymphoma (FL) transformation, many patients are diagnosed with transformation by clinical factors as biopsy specimens often cannot be obtained. Despite the frequency of clinical diagnosis, no clinical assessment tool has yet been established for FL transformation in the rituximab era. We derived and validated a transformation scoring system (TSS) based on retrospective analyses of 126 patients with biopsy‐proven FL and histologic transformation (HT) at two hospitals of the National Cancer Center of Japan. In the derivation set (76 patients), the detailed analyses of the clinical characteristics at disease progression showed that lactate dehydrogenase (LDH) elevation, focal lymph nodal (LN) enlargement, hemoglobin <12 g/dl, and poor performance status (PS) (2‐4) were associated with HT. The weights of these variables were decided based on the regression coefficients. Next, we constructed a TSS encompassing the above four factors: LDH, (> upper limit of normal [ULN], ≤ULN ×2) (1 point), (≥ULN ×2) (2 points); focal LN enlargement, (≥3 cm, <7 cm) (1 point), (≥7 cm) (2 points); hemoglobin <12 g/dl (1 point); poor PS (2 points). We identified a high positive predictive value (PPV) (96.4%) and negative predictive value (NPV) (85.4%) for diagnosing HT when a cutoff score of 2 was selected for our TSS. In an external validation set (50 patients), the probability of HT was high with scores ≥2 (PPV, 93.3%; NPV, 82.9%). We developed a TSS that offers a simple, yet, valuable tool, for diagnosing HT, especially in patients who cannot undergo biopsy.

## Graphical abstract

We developed a new transformation scoring system (TSS) for determining the clinical transformation of FL and successfully validated it in an independent cohort. TSS promises to be a simple, yet valuable tool, for the diagnosis of clinical transformation in both daily practice and clinical trials, especially in patients for whom obtaining a biopsy specimen is not feasible.

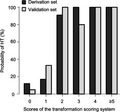

## 1 INTRODUCTION

Follicular lymphoma (FL) is a major type of indolent B‐cell lymphoma. The watch and wait (WW) strategy remains an acceptable approach for FL patients, especially for those with low tumor burden, even in the rituximab era.^1^, ^2^, ^3^, ^4^, ^5^ Although FL remains an incurable disease, clinical outcomes, even in patients with high tumor burden, have improved, mainly owing to chemoimmunotherapy.^1^, ^13^

However, histologic transformation (HT) is a critical event because the prognosis of FL patients with HT is poorer than that of FL patients without HT; this has remained true even after the introduction of rituximab.^14^, ^15^, ^16^, ^17^, ^18^, ^19^, ^20^, ^21^ Several studies have reported that transformation from FL to aggressive lymphoma occurs in 4%–22% of the patients within 5 years from the initial diagnosis.^15^, ^16^, ^17^, ^18^, ^26^ The probability of transformation varies among these studies owing to the heterogeneity in study designs, such as the inclusion criteria of patients (especially if those with clinical transformation were included^20^, ^22^, ^24^, ^26^ or excluded^16^, ^17^, ^18^, ^21^, ^23^, ^25^), the years when the studies were conducted (in the pre‐rituximab era,^22^, ^23^, ^24^, ^25^ or in the rituximab era^15^, ^16^, ^17^, ^18^, ^20^, ^21^, ^26^), and the definition of transformation.

Although histologic confirmation by biopsy is the gold standard for diagnosing transformation,^27^ it is not always possible to obtain the specimen for biopsy (e.g., in cases when disease progression is in an inaccessible location or develops very rapidly). Of note, even in prospective studies,^16^, ^21^, ^28^ the specimen for biopsy could not be obtained in 60%–80% of the patients at the time of disease progression. Moreover, a previous study has reported that more than half of the FL patients with transformation were diagnosed based only on clinical criteria and at the physician's discretion without a histologic confirmation.^15^ Moreover, limited information is available regarding the clinical factors at the time of disease progression that are associated with the transformation.^17^, ^18^, ^23^, ^24^ Although the clinical criteria for transformation had been proposed in the pre‐rituximab era,^24^ a recent retrospective study indicated that such criteria may not be reliably accepted in the rituximab era.^18^ Moreover, to the best of our knowledge, no study has been conducted in the rituximab era to compare and statistically identify the clinical factors associated with disease progression in patients with biopsy‐proven FL and HT.

Therefore, the present study conducted a retrospective analysis at two hospitals of the National Cancer Center of Japan to develop a transformation scoring system (TSS) for the diagnosis of the clinical transformation of FL that would be easy to use in both daily practice and clinical trials.

## 2 MATERIALS AND METHODS

### 2.1 Study design

This retrospective study utilized derivation and validation patient cohorts to develop definition criteria for FL clinical transformation. Patients initially diagnosed with FL (grades 1, 2, or 3a) according to the World Health Organization's classification^29^, ^30^ were included. Patients with grade 3b FL and composite lymphoma (i.e., confirmed to have both FL and diffuse large B‐cell lymphoma [DLBCL]) at initial diagnosis were excluded.

To assess the definition of clinical transformation of FL, we retrospectively analyzed patients who were initially diagnosed with FL (grades 1, 2, or 3a) and underwent biopsy at the time of disease progression at the National Cancer Center Hospital (NCCH) between 2000 and 2016. Using this cohort of patients (the derivation cohort), we investigated the clinical characteristics at the time of disease progression and constructed a TSS based on clinical covariates obtained by multivariate logistic regression model.

To validate the TSS, we retrospectively analyzed two cohorts of patients who were initially diagnosed with FL (grades 1, 2, or 3a). First cohort comprised patients who did not undergo biopsy at the time of disease progression and who were diagnosed at the NCCH between 2000 and 2016 (the internal validation cohort). Second cohort comprised patients who underwent biopsy at the time of disease progression and were diagnosed at the NCCH‐East (NCCHE) as a completely independent cohort between 2003 and 2014 (the external validation cohort). We applied the TSS to both cohorts.

This study was approved by the Institutional Review Board of the National Cancer Center and was conducted in accordance with the principles of Declaration of Helsinki.

### 2.2 Definition of transformation

HT was defined based on biopsy confirmation involving both an increase in the number of large cells and a loss of follicular structure. Progression from grade 1 and 2 to grade 3 was not included in HT. Only biopsy‐proven transformation from FL to DLBCL was included as HT; transformations from FL to other histological types (Burkitt or Hodgkin lymphoma) were excluded.

### 2.3 Statistical analyses

Categorical variables were compared using the Fisher's exact test. The probability of overall survival (OS) was calculated using the Kaplan‐Meier method, and the groups were compared using the log‐rank test. The OS from disease progression was defined as the duration from disease progression to death from any cause or the date of the last follow‐up. The cumulative incidence of HT was calculated using the Gray's method. In a competing risk model for HT, death before HT was defined as a competing risk. The time to HT was calculated as the duration between the date of initial diagnosis of FL and the occurrence of HT. Clinical data for each patient were extracted from the patient's medical records. A two‐sided *p*‐value <0.05 was considered statistically significant. Variables significantly associated with HT in univariate analysis were included in the multivariate logistic regression model. Clinical stage was determined according to the Ann Arbor classification system. Focal lymph nodal (LN) enlargement was defined when the nodal mass larger than 3 cm was observed in only one nodal area and the size of nodal masses in other nodal areas was less than 3 cm. The nodal area was defined according to the Follicular Lymphoma International Prognostic Index (FLIPI)^31^. Focal LN enlargement was also assessed for larger diameter (the nodal mass ≥7 cm). Bulky disease was defined as the nodal mass ≥6 cm in diameter, regardless of the number of nodal areas. The maximum standardized uptake value (SUVmax) was assessed for patients who received ^18^F‐fluorodeoxyglucose positron emission tomography/computed tomography (FDG‐PET/CT). The TSS scores were calculated from a regression coefficient for each statistically significant variable. Receiver operating characteristic (ROC) curve analysis was used to assess the accuracy of the TSS and SUVmax, the cutoff values for which were determined with a high positive predictive value (PPV) and negative predictive value (NPV). Statistical analyses were performed using the EZR software package, version 1.32 (Saitama Medical Center, Jichi Medical University, Saitama, Japan), which is a graphical user interface for R (The R Foundation for Statistical Computing, version 3.2.4).^32^

## 3 RESULTS

### 3.1 Development of the transformation scoring system in the derivation set

**FIGURE 1:**
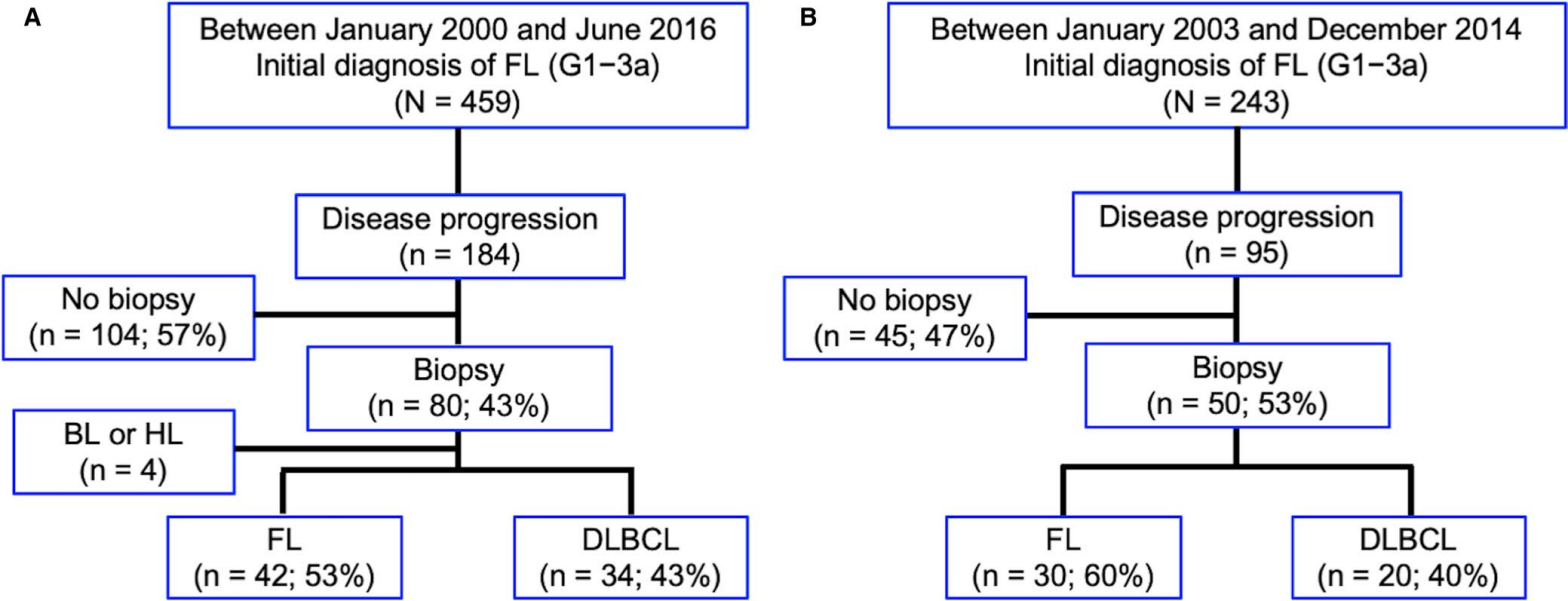
Flowcharts for patients’ selection The NCCH cohort (A) and the NCCHE cohort (B). Abbreviations: NCCH, National Cancer Center Hospital; NCCHE, National Cancer Center Hospital‐East; FL, follicular lymphoma; BL, Burkitt lymphoma; HL, Hodgkin lymphoma; DLBCL, diffuse large B‐cell lymphoma

Patients’ selection flowcharts are shown in Figure 1A‐B. During the study period in the NCCH cohort, 459 patients were diagnosed with FL (grades 1, 2, or 3a) at the NCCH (Figure 1A). The median duration of follow‐up among these patients was 7.1 (range: 0.2‐16.6) years. Disease progression was observed in 184 patients, among whom 80 (43%) had the histologic documentation (FL in 42, HT with DLBCL in 34, and HT other than DLBCL in 4). Finally, we identified 76 patients with biopsy‐proven FL or HT with DLBCL as subjects for the derivation analysis. In this cohort, the first‐line treatment between FL and HT was similar; 22 patients (28.9%; FL in 11 and HT in 11) were initially managed with WW, 45 patients (59.2%, FL in 24 and HT in 21) were immediately treated with rituximab‐containing therapy, and nine patients (11.8%, FL in 7 and HT in 2) were immediately treated with local radiotherapy. Further, both groups had similarly received R‐CHOP therapy before disease progression (FL in 20 and HT in 20).

**FIGURE 2:**
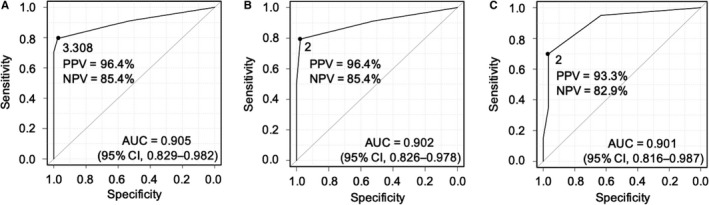
Receiver operating characteristic curve analysis of the transformation scoring system Before (A) and after (B) the simplification of the TSS scores in the derivation set, and in the external validation set (C). Abbreviations: TSS, transformation scoring system; AUC, area under the receiver operating characteristic curve; CI, confidence interval; PPV, positive predictive value; NPV, negative predictive value.

The clinical characteristics of 76 patients with biopsy‐proven FL or HT at the time of disease progression are shown in Table 1. The median age was 61.5 years (range, 32‐85 years). On univariate analysis, B symptoms, poor Eastern Cooperative Oncology Group performance status (PS) score (2‐4), lactate dehydrogenase (LDH) level over twice the upper limit of normal (ULN), focal LN enlargement (≥7 cm), and hypercalcemia at disease progression were observed only in patients with HT. More patients in the HT group had LDH elevation, hemoglobin <12 g/dl, C‐reactive protein elevation, bulky disease, and focal LN enlargement (≥3 cm). On multivariate analysis, poor PS score, focal LN enlargement, LDH elevation, and hemoglobin <12 g/dl at disease progression were associated with HT (Table 2). We constructed the scoring system consisting of the abovementioned four factors; the weights of the variables were decided based on the regression coefficients. To assess the cutoff value that best distinguished HT from FL, we used the ROC curve analysis (Figure 2A). The area under the ROC curve (AUC) was high (0.91, 95% confidence interval [CI]: 0.828‐0.981); the cutoff score was determined to be 3.31, which produced a high PPV (96.4%) and NPV (85.4%).

**TABLE 1:** Clinical characteristics of the biopsy‐proven follicular lymphoma (FL) or histologic transformation (HT) patients at the time of disease progression in the derivation set [Correction added on 22 October 2020, after first online publication: In Table 1, the calculation of the total number of patients (FL patients + HT patients) in the "Number of relapses from initial diagnosis" has been corrected from "14 (18.4)" to "23 (30.3)" in this version]

Characteristics	Total (N = 76)		FL (n = 42)		HT (n = 34)		*p*‐value^a^
	No.	%	No.	%	No.	%	
Age							
Median (range), years	61.5 (32‐85)		62.5 (36‐85)		61 (31‐83)		0.493
<61	33	43.4	17	40.5	16	47.1	0.644
≥61	43	56.6	25	59.5	18	52.9	
Sex							
Female	38	50.0	17	40.5	21	61.8	0.106
Male	38	50.0	25	59.5	13	38.2	
B symptoms							
No	65	85.5	39	92.9	26	76.5	0.003
Yes	7	9.2	0	0.0	7	20.6	
Missing	4	5.3	3	7.1	1	2.9	
ECOG performance status							
0‐1	65	85.5	40	95.2	25	73.5	0.001
2‐4	8	10.5	0	0.0	8	23.5	
Missing	3	3.9	2	4.8	1	2.9	
Ann Arbor Stage							
I‐II	19	25.0	11	26.2	8	23.5	0.585
III‐IV	45	59.2	21	50.0	24	70.6	
Missing	12	15.8	10	23.8	2	5.9	
LDH							
Median (range), IU/L	197 (116‐5540)		176.5 (116‐288)		276.5 (143‐5540)		<0.001
≤ULN	46	60.5	35	83.3	11	32.4	<0.001
>ULN	30	39.5	7	16.7	23	67.6	
≤ULN ×2	67	88.2	42	100.0	25	73.5	<0.001
>ULN ×2	9	11.8	0	0.0	9	26.5	
Hemoglobin							
Median (range), g/dl	12.85 (4‐17.4)		13.15 (9.8‐17.4)		12.55 (4‐15.2)		0.011
<12	20	26.3	6	14.3	14	41.2	0.010
≥12	56	73.7	36	85.7	20	58.8	
White blood cell count							
Median (range), /μl	5100 (800‐40200)		5100 (2900‐40200)		5050 (800‐31000)		0.415
Platelet count							
Median (range), ×10^4^ /μl	17.8 (0.3‐51.1)		18.15 (8.9‐39.8)		17.55 (0.3‐51.1)		0.758
Hypercalcemia							
Median (range), mg/dl	9.4 (7.7‐12)		9.4 (8.510.3)		9.4 (7.7‐12)		0.806
No	74	97.4	42	100.0	32	94.1	0.197
Yes	2	2.6	0	0.0	2	5.9	
CRP							
Median (range), mg/dl	0.155 (0.02‐26.9)		0.1 (0.02‐4.34)		0.705 (0.02‐26.9)		0.001
≤ULN	36	47.4	25	59.5	11	32.4	0.022
>ULN	40	52.6	17	40.5	23	67.6	
Bone marrow involvement							
Negative	41	53.9	22	52.4	19	55.9	0.355
Positive	14	18.4	5	11.9	9	26.5	
Missing	21	27.6	15	35.7	6	17.6	
Extranodal site, excluding BM							
Negative	50	65.8	31	73.8	19	55.9	0.080
Positive	24	31.6	9	21.4	15	44.1	
Missing	2	2.6	2	4.8	0	0.0	
Bulky disease							
Median (range), cm	3.3 (0‐12.1)		2.8 (0‐8.2)		5.6 (0‐12.1)		<0.001
<6 cm	57	75.0	39	92.9	18	52.9	<0.001
≥6 cm	18	23.7	2	4.8	16	47.1	
Missing	1	1.3	1	2.4	0	0.0	
Focal lymph nodal enlargement							
No^b^	47	61.8	33	78.6	14	41.2	0.001
Yes (≥3 cm)^c^	27	35.5	8	19.0	19	55.9	
No^d^	64	84.2	41	97.6	23	67.6	<0.001
Yes (≥7 cm)^e^	10	13.2	0	0.0	10	29.4	
Missing	2	2.6	1	2.4	1	2.9	
SUVmax							
Median (range)	11.63 (2.11‐33.34)		9.20 (2.11‐16.7)		16.74 (4.86‐33.34)		<0.001
Missing	26	34.2	14	33.3	12	35.3	1.00
FDG‐PET/CT	50	65.8	28	66.7	22	64.7	
SUVmax <10	19	38.0	16	57.0	3	13.6	0.003
SUVmax ≥10	31	62.0	12	43.0	19	86.4	
SUVmax <16	36	72.0	27	96.4	9	40.9	<0.001
SUVmax ≥16	14	28.0	1	3.6	13	59.1	
SUVmax <20	45	90.0	28	100.0	17	77.3	0.012
SUVmax ≥20	5	10.0	0	0.0	5	22.7	
FLIPI							
Low risk	26	34.2	18	42.9	8	23.5	0.046
Intermediate risk	19	25.0	11	26.2	8	23.5	
Poor risk	28	36.8	10	23.8	18	52.9	
Missing	3	3.9	2	4.8	1	2.9	
IPI							
Low risk	27	35.5	19	45.2	8	23.5	<0.001
Low‐intermediate risk	27	35.5	18	42.9	9	26.5	
High‐intermediate risk	12	15.8	3	7.1	9	26.5	
High risk	7	9.2	0	0.0	7	20.6	
Missing	3	3.9	2	4.8	1	2.9	
Number of relapses from initial diagnosis							
1	53	69.7	33	78.6	20	58.8	0.081
≥2	23	30.3	9	21.4	14	41.2	

Abbreviations: BM, bone marrow; CRP, C‐reactive protein; ECOG, Eastern Cooperative Oncology Group; FDG‐PET/CT,^18^F‐fluorodeoxyglucose positron emission tomography/computed tomography; FLIPI, Follicular Lymphoma International Prognostic Index; IPI, Internal Prognostic Index; LDH, lactate dehydrogenase; SUVmax, maximum standardized uptake value; ULN, upper limit of normal.

^a^ *p*‐value was analyzed by comparing the biopsy‐proven FL patients with HT patients.

^b^ Not applicable to ^c^.

^c^ The nodal mass (≥3 cm) was observed in only one nodal area.

^d^ Not applicable to ^e^,

^e^ The nodal mass (≥7 cm) was observed in only one nodal area.

**TABLE 2:** Multivariate analysis and scores of the transformation scoring system

Risk factors		Regression coefficients	Scores
LDH	>ULN, ≤ULN ×2	1.7733	1
	≥ULN ×2	20.1753	2
Focal lymph nodal enlargement	≥3 cm, <7 cm	1.7733	1
	≥7 cm	20.0174	2
Hemoglobin	<12 g/dl	1.5352	1
PS	2−4	19.7186	2

Abbreviations: LDH, lactate dehydrogenase; PS, performance status; ULN, upper limit of normal.

**FIGURE 3:**
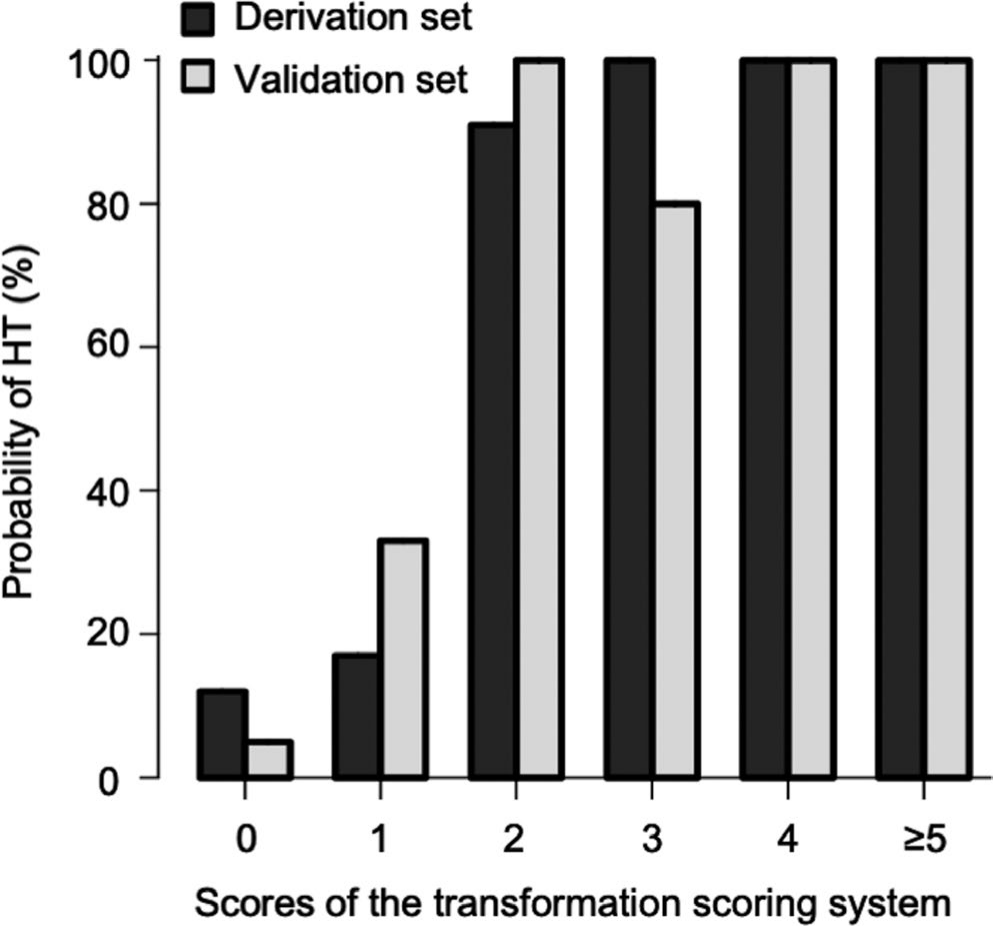
Probability of histologic transformation according to the transformation scoring system in the derivation and external validation sets Abbreviation: HT, histologic transformation

To develop a simple scoring system so as not to change the previous PPV/NPV, we assigned the scores with reference to the regression coefficients and the previous cutoff score (Table 2). Using the simplified transformation scoring system, TSS, the cutoff value was identified to be 2, which gave the same predictive value; the PPV and NPV were 96.4% and 85.4%, respectively (Figure 2B). According to the TSS score, the percentage of HT patients with scores of 0, 1, 2, 3, 4, and ≥5 were 12%, 17%, 91%, 100%, 100%, and 100%, respectively (Table 3 and Figure 3).

**TABLE 3:** Distribution of patients stratified by the transformation scoring system

TSS, scores	Derivation set				External validation set			
	Total	FL	HT	Probability of HT	Total	FL	HT	Probability of HT
	(N = 76)	(n = 42)	(n = 34)	(%)	(N = 50)	(n = 30)	(n = 20)	(%)
0	25	22	3	12	20	19	1	5
1	23	19	4	17	15	10	5	33
2	11	1	10	91	7	0	7	100
3	9	0	9	100	5	1	4	80
4	4	0	4	100	2	0	2	100
≥5	4	0	4	100	1	0	1	100

Abbreviations: FL, follicular lymphoma; HT, histologic transformation; TSS, transformation scoring system.

### 3.2 External validation set

As shown in Figure 1B (the NCCHE cohort), 243 patients were diagnosed with FL (grades 1, 2, or 3a) at the NCCHE with a median follow‐up duration of 5.7 (range: 0.1‐14.4) years. Disease progression was observed in 95 patients, of whom 50 (53%) had the histologic documentation (FL in 30 and HT with DLBCL in 20). Finally, we identified 50 patients with biopsy‐proven FL or HT with DLBCL as subjects for the external validation analysis.

The clinical characteristics of 50 patients with biopsy‐proven FL or HT at the time of disease progression are shown in Table S1. We applied the TSS to this completely independent cohort for external validation. Based on the ROC curve analysis, the AUC of the TSS in the external validation cohort was 0.900 (95% CI, 0.815‐0.987) (Figure 2C). Furthermore, a score of 2 or higher produced a high PPV and NPV of 93.3% and 82.9%, respectively, for HT diagnosis, which confirmed the validity of the TSS. According to the TSS score, the percentage of patients with HT who had scores of 0, 1, 2, 3, 4, and ≥5 were 5%, 33%, 100%, 80%, 100% and 100%, respectively (Table 3 and Figure 3).

### 3.3 Outcomes and internal validation set

Among 459 patients with FL in the NCCH cohort, HT occurred at a median of 5.5 years (range, 0.2‐16 years) after the initial FL diagnosis; the cumulative incidences of HT at 5 and 10 years were 4.2% (95% CI, 2.5‐6.6) and 8.5% (95% CI, 5.6‐12.1), respectively (Figure S1A). Among 243 patients with FL in the NCCHE cohort, HT occurred at a median of 5.3 years (range, 0.7‐12.8 years) after the initial FL diagnosis, and the cumulative incidences of HT at 5 and 10 years were 5.0% (95% CI, 2.5‐8.7) and 13.3% (95% CI, 7.7‐20.3), respectively (Figure S1B).

**FIGURE 4:**
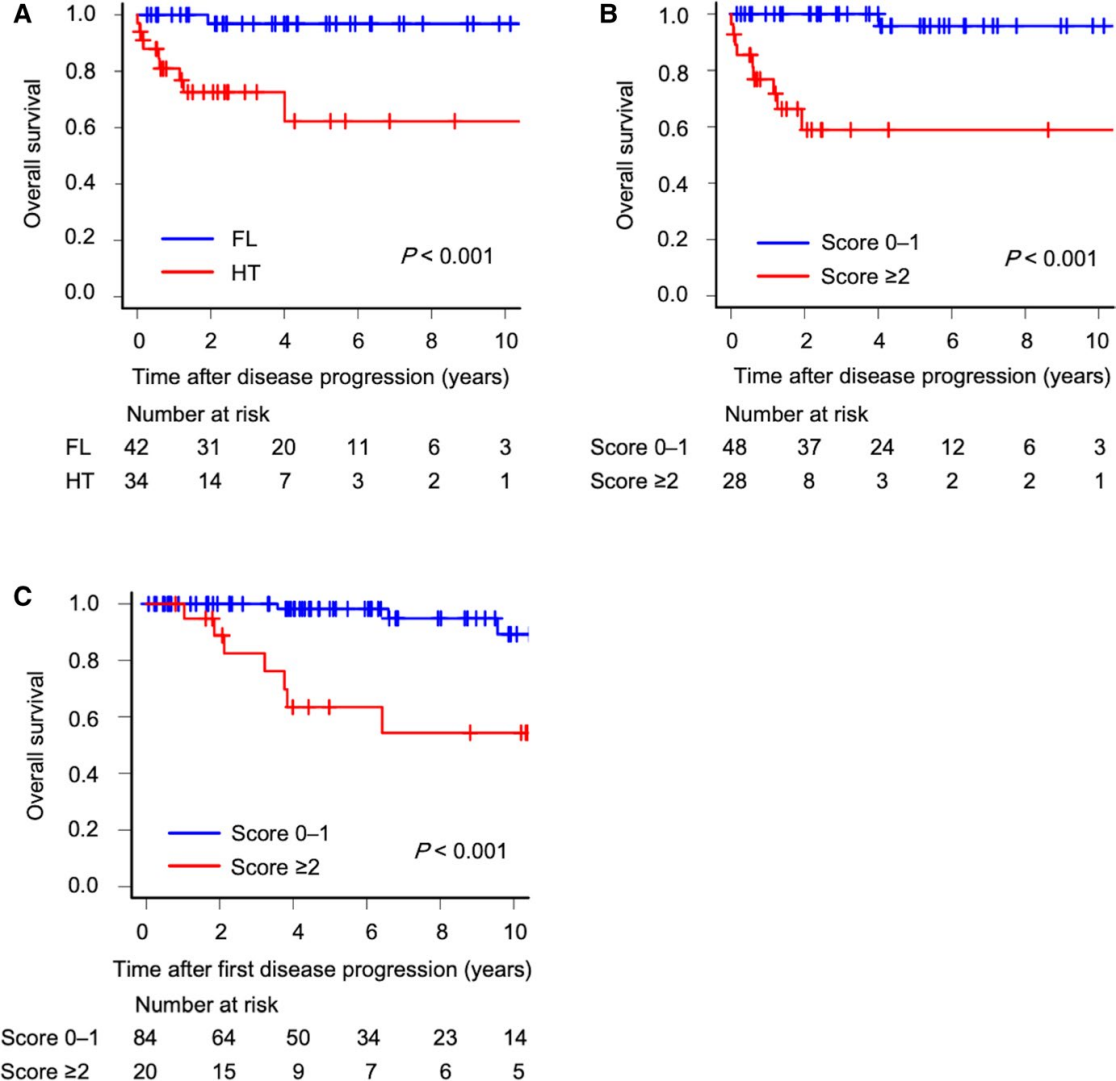
Kaplan‐Meier curves showing overall survival according to histology and the transformation scoring system Probability of overall survival after disease progression in patients with FL vs. HT in the derivation set (A), overall survival after disease progression in patients with high vs. low TSS scores in the derivation set (B), and overall survival after disease progression in 104 patients who did not undergo biopsy at the first progression with high vs. low TSS scores in the internal validation set (C). Abbreviations: FL, follicular lymphoma; HT, histologic transformation; TSS, transformation scoring system

Among 76 patients with biopsy‐proven FL or HT in the derivation set, the probability of 5‐year OS after disease progression was 96.9% (95% CI, 79.8‐99.6) in patients with biopsy‐proven FL and 62.2% (95% CI, 34.9‐80.8) in patients with HT (*p* < 0.001; Figure 4A). Further, the probability of 5‐year OS after disease progression was lower in patients with higher TSS scores (≥2) than in patients with lower scores (0‐1) (58.9% [95% CI, 34.0‐77.1] vs. 95.8% [95% CI, 73.9‐99.4], *p* < 0.001; Figure 4B).

Furthermore, among the 459 patients with FL in the NCCH cohort, 104 who developed disease progression could not undergo biopsy, including seven who were diagnosed with clinical transformation and treated accordingly. As an internal validation analysis, the TSS score distributions at first disease progression in these 104 patients are shown in Figure S1C and Table S2; 20 patients (19%) had a score of 2 or higher. Interestingly, the probability of 5‐year OS after disease progression was lower in patients with higher scores than in patients with lower scores (63.4% [95% CI, 35.8‐81.7] vs. 98.2% [95% CI, 88.0‐99.7], *p* < 0.001; Figure 4C). Further, almost all the patients (86%) with higher TSS scores died of lymphoma, as shown in Table S2. Regarding the salvage therapies for the patients with higher scores, there were no statistically significant differences between this cohort and the derivation cohort, except with rituximab monotherapy (Table S3).

## 4 DISCUSSION

We developed and validated a new scoring system for determining the clinical transformation of FL, TSS, using two independent cohorts (the NCCH and NCCHE cohorts). It is difficult to obtain the specimen for biopsy in all patients with FL at the time of disease progression. In fact, the previous studies have indicated a low rate (20.6‐42%) of performing biopsy at the time of disease progression of FL.^16^, ^21^, ^28^ Therefore, although histologic analysis is the gold standard for diagnosing transformation, our new scoring system will be useful for assessing the probability of transformation in patients who are unable to undergo the biopsy.

Diagnosing transformation is important for patients with FL because, despite the availability of rituximab, HT is still strongly associated with mortality in patients with FL.^19^, ^21^ Further, treatment strategies for patients with HT could be more intensive than those for patients without HT, and include procedures such as hematopoietic stem cell transplantation.^16^, ^33^, ^34^, ^35^ Moreover, because the incidence of HT is one of the designated clinical trial endpoints of FL, reliable diagnosis of transformation is essential for assessing this endpoint accurately. However, there are many patients with FL who could not undergo a biopsy at the time of disease progression, even in clinical trials,^15^, ^16^, ^20^, ^23^, ^24^, ^26^ which resulted in varying rates of transformation reported among such trials. Patients without biopsies are currently diagnosed with clinical transformation solely based on their clinical characteristics; however, because of the lack of standardized criteria for diagnosing the clinical transformation of FL, it has been difficult to compare the incidence rate of HT among the previously published studies.

Several studies have compared the clinical factors of FL and HT at the time of the initial diagnosis of FL to predict the risk of HT,^15^, ^16^, ^17^, ^18^, ^26^ including prospective cohort studies with a large number of patients.^16^, ^26^ However, among available studies, the number of HT patients diagnosed by biopsy were limited,^16^, ^17^, ^18^, ^21^, ^23^, ^25^ which resulted in varying HT risk factors and incidence rates being reported. On the contrary, although there have been a few studies that assessed the clinical factors at the time of disease progression in patients with HT,^17^, ^18^, ^23^, ^24^ detailed comparisons between FL and HT have not been performed in the era of rituximab availability. Therefore, we elucidated the clinical factors associated with HT at the time of disease progression in the immunochemotherapy era.

A well‐known criterion for clinical transformation has been derived from the Vancouver population‐based analysis in the pre‐rituximab era,^24^ wherein the clinical transformation was arbitrarily defined as exhibiting one or more of the five clinical manifestations including rapid nodal growth, extranodal sites, new B symptoms, LDH over twice the ULN, and new hypercalcemia. The reliability of this criterion was demonstrated by the close similarity in the clinical outcomes of patients diagnosed with clinical transformation using the criterion, to those diagnosed by biopsy. However, cohorts of patients with HT may be different in the pre‐ and post‐rituximab eras, since the comparisons of these two periods have shown that the clinical outcomes of patients with HT were worse and the incidence of HT was higher in the pre‐rituximab era.^15^, ^18^, ^21^, ^26^ In addition, these five clinical factors were not verified using statistical models, although the impact of each of these factors on patients with HT is likely to be different. Thus, currently, there are no standardized criteria for diagnosing the clinical transformation of FL. In our study, we extracted the detailed clinical factors at the time of disease progression only from patients with biopsy‐proven histology and performed statistical analyses, including the validation analysis, on these factors. This was in an attempt to standardize the definition of clinical transformation in the rituximab era. Furthermore, as one of the factors, “rapid nodal growth,” comprising the Vancouver criterion was not rigorously defined, it may be difficult to accurately use this criterion in both daily practice and clinical trials. In contrast, the TSS, comprising of “focal LN enlargement,” was strictly defined and may indicate that only one nodal area progressed more rapidly than other nodal areas in patients with HT, which might better describe “rapid nodal growth.” Another possibility is that the persistence of one enlarged LN in FL patients may be associated with the development of HT. Therefore, the TSS can be evaluated quantitatively at a single time, thereby providing easy access to FL transformation in both daily practice and clinical trials.

As an internal validation analysis, we applied the TSS to 104 patients who did not undergo biopsy at the time of disease progression. Among them, the majority of patients (81%) had lower scores, according to the TSS. Importantly, the prognosis of the patients with higher scores (n = 20) was similar to that of patients with HT, although the salvage therapies among both cohorts were not the same. This might indicate that the TSS can be used to stratify FL patients with disease progression who did not undergo biopsy, and can diagnose them with the clinical transformation. Moreover, our new scoring system may be used as a prognostic index at the time of FL disease progression because among the 180 patients with disease progression in the NCCH cohort, the prognoses of patients with higher scores were poorer than that in the patients with lower scores, as shown in Figure 4B,C.

We also assessed the SUVmax value to distinguish HT patients from FL patients who underwent FDG‐PET/CT (Supplementary material). In the both derivation and validation cohorts, a high SUVmax value indicated that patients with FL had developed HT, which was consistent with the previous studies.^36^, ^37^, ^38^, ^39^ Although we tried to incorporate the SUVmax value into the TSS, a superior model could not be developed. Even in the cohort of patients who received PET/CT, the TSS was superior to the scoring system, which incorporated the SUVmax value in the derivation set (data not shown). Furthermore, the scoring system with the incorporated SUVmax value was not validated well because the SUVmax value in patients with HT in the external validation cohort was higher than that in the derivation cohort despite using similar PET/CT scanner, protocol, and software in the hospitals. Theoretically, the SUVmax value would vary among institutions because of the difference in PET/CT scanner and the method of SUV quantification. Thus, it is difficult to apply a certain SUVmax value to other institutions. In addition, because a recent study suggested that a high SUVmax value of the patients with FL at initial diagnosis was not associated with HT,^40^ an increase in SUVmax value at the time of disease progression compared to that at initial diagnosis may be important to assess HT. Owing to the aforementioned reasons, we did not incorporate the SUVmax value in the TSS.

This study has several limitations. First, due to the retrospective nature of this study, we analyzed limited number of patients who underwent a biopsy at the time of disease progression. This might have resulted in potential bias in developing the TSS, even though we validated it in a completely independent cohort. Second, the decision to perform a biopsy was at the physician's discretion; however, the TSS was also validated in patients who did not undergo biopsy at the first progression. Third, as the TSS was developed to assess HT at the time of disease progression, it cannot predict HT in patients who were initially diagnosed with FL. Therefore, to confirm the TSS, prospective studies comprising a large number of patients may be warranted.

In conclusion, we developed a new scoring system for the clinical transformation of FL, TSS, and validated it in an independent cohort. The TSS promises to be a simple, yet, valuable tool, for the diagnosis of clinical transformation in both daily practice and clinical trials, especially in patients for whom obtaining a biopsy specimen is not feasible.

## CONFLICT OF INTEREST

DM has received research funding from Celgene, Chugai Pharmaceutical, Janssen Pharmaceutical, MSD, Mundipharma, Novartis Pharma, Ono Pharmaceutical, and Takeda Pharmaceutical and honoraria from Celgene, Chugai Pharmaceutical, Eisai, Janssen Pharmaceutical, Kyowa Kirin, Mundipharma, and Takeda Pharmaceutical. NY has received research funding from Amgen, Celgene, Ono Pharmaceutical, and paid expert testimony from Takeda Pharmaceutical. KI has received research funding from AbbVie, AstraZeneca, Bayer Pharma, Celgene, Chugai Pharmaceutical, Daiichi Sankyo, Eisai, HUYA Japan, Kyowa Kirin, Incyte, Janssen Pharmaceutical, Novartis Pharma, Ono Pharmaceutical, Pfizer, Sanofi, Solasia Pharma, Symbio, Takeda Pharmaceutical, Yakult, and Zenyaku Kogyo, and honoraria from Eisai, and Kyowa Kirin. KeT has received honoraria from Celgene, Chugai Pharmaceutical, Daiichi Sankyo, Eisai, HUYA Bioscience International, Kyowa Kirin, Mundi Pharma, Ono Pharmaceutical, Takeda Pharmaceutical, Yalult, and Zenyaku Kogyo. All other authors declare no conflict of interest.

## AUTHORS’ CONTRIBUTIONS

Conception and design: Takafumi Shichijo, Dai Maruyama Provision of study materials or patients: All authors Collection and assembly of data: Takafumi Shichijo, Nobuhiko Yamauchi, Sayako Yuda, Akiko Miyagi Maeshima, Masato Sugano, Hiroaki Kurihara, Kaoru Shimada, Dai Maruyama Data analysis and interpretation: Takafumi Shichijo, Dai Maruyama, Kinuko Tajima, Koji Izutsu, Kensei Tobinai Manuscript writing: Takafumi Shichijo, Dai Maruyama Final approval of manuscript: All authors

## Supporting information

Supplementary Material

## Data Availability

Inquiries for data should be directed to dmaruyam@ncc.go.jp. The date will be available for achieving aims in the approved proposal.
