# Supplementary material for: Transformation Scoring System (TSS): A new assessment index for clinical transformation of follicular lymphoma
Source: Cancer Med. 2020 Oct 6;9(23):8864–74. doi: 10.1002/cam4.3501 (PMC7724492; doi:10.1002/cam4.3501)
Supplement: Supplementary file 1 — Supplementary Material [file CAM4-9-8864-s001.doc]

**Supplementary material**

**^18^F-fluorodeoxyglucose positron emission tomography/computed tomography (FDG-PET/CT) for the diagnosis of clinical transformation**

In the derivation set, 50 among the 76 patients (66%) received FDG-PET/CT at the time of disease progression. The site of maximum standardized uptake value (SUVmax) measurement was essentially the same as that of the biopsy. The median SUVmax was 9.20 (range, 2.11–16.70) in patients with follicular lymphoma (FL) (n = 28) and 16.74 (range, 4.86–33.34) in patients with histologic transformation (HT) (n = 22) (*P* < 0.001; **Table 1 and Figure S2A**). We used the receiver operating characteristic (ROC) analysis to assess the SUVmax cut-off that distinguished HT from FL (**Figure S2B**). The SUVmax was found to be a useful parameter for distinguishing HT from FL, as the area under the ROC curve (AUC) was high (0.830 [95% confidence interval {CI}, 0.715–0.946]). SUVmax cut-off values of 10.0, 16.0, and 20.0 produced positive predictive values (PPVs) of 61.3%, 92.9%, and 100.0%, respectively; and negative predictive values (NPVs) of 84.2%, 75.0%, and 62.2%, respectively.

In the validation set, 28 among 50 patients (56%) underwent FDG-PET/CT at the time of disease progression and the median SUVmax was 12.23 (range, 2.61–20.33) in patients with FL (n = 19) and 27.48 (range, 10.41–46.6) in patients with HT (n = 9) (*P* = 0.007; **Table S1 and Figure S2C**). The AUC was 0.813 (95% CI, 0.620–1.000) (**Figure S2D**). The SUVmax cut-off values of 10.0, 16.0, and 20.0 produced PPVs of 42.9%, 60.0%, and 85.7%, respectively; and NPVs of 100.0%, 83.3%, and 85.7%, respectively.

**Supplementary Figure S1. Cumulative incidence of histologic transformation in the derivation and external validation sets, and score distributions of patients stratified by the transformation scoring system in the internal validation cohort**

Cumulative incidence of HT in the NCCH cohort (A) and the NCCHE cohort (B). The score distribution according to the TSS in the internal validation set (C).

Abbreviations: NCCH, National Cancer Center Hospital; NCCHE, National Cancer Center Hospital-East; HT, histologic transformation; FL, follicular lymphoma; TSS, transformation scoring system.

**Supplementary Figure S2. Box plots and receiver operating characteristic curve analysis of the maximum standardized uptake value**

Box plots of SUVmax, comparing patients of FL with HT in the NCCH cohort (A) and NCCHE cohort (C). Receiver operating characteristic curve analysis of SUVmax in the NCCH cohort (B) and NCCHE cohort (D). Abbreviations: NCCH, National Cancer Center Hospital; NCCHE, National Cancer Center Hospital-East; FL, follicular lymphoma; HT, histologic transformation; SUVmax, maximum standardized uptake value.

Table S1. Clinical characteristics of the biopsy-proven follicular lymphoma (FL) or histologic transformation (HT) patients at the time of disease progression in the external validation cohort

|  |  | Total (N = 50) | |  | FL (n = 30) | |  | HT (n = 20) | |  |
| --- | --- | --- | --- | --- | --- | --- | --- | --- | --- | --- |
| Characteristics | | No. | % |  | No. | % |  | No. | % | *P*-value^a^ |
| Age | |  |  |  |  |  |  |  |  |  |
|  | median (range), years | 64.5 (40−86) | |  | 66 (40−79) | |  | 62 (44−86) | | 0.788 |
|  | <61 | 22 | 44.0 |  | 12 | 40.0 |  | 10 | 50.0 | 0.567 |
|  | ≥61 | 28 | 56.0 |  | 18 | 60.0 |  | 10 | 50.0 |  |
| Sex | |  |  |  |  |  |  |  |  |  |
|  | Female | 30 | 60.0 |  | 19 | 63.3 |  | 11 | 55.0 | 0.572 |
|  | Male | 20 | 40.0 |  | 11 | 36.7 |  | 9 | 45.0 |  |
| ECOG performance status | |  |  |  |  |  |  |  |  |  |
|  | 0−1 | 45 | 90.0 |  | 30 | 100.0 |  | 15 | 75.0 | 0.126 |
|  | 2−4 | 2 | 4.0 |  | 0 | 0.0 |  | 2 | 10.0 |  |
|  | Missing | 3 | 6.0 |  | 2 | 6.7 |  | 3 | 15.0 |  |
| B symptoms | |  |  |  |  |  |  |  |  |  |
|  | No | 46 | 92.0 |  | 30 | 100.0 |  | 16 | 80.0 | 0.021 |
|  | Yes | 4 | 8.0 |  | 0 | 0.0 |  | 4 | 20.0 |  |
| LDH | |  |  |  |  |  |  |  |  |  |
|  | median (range), IU/L | 225 (117−639) | |  | 206.5 (117−596) | |  | 302 (121−639) | | <0.001 |
|  | ≤ULN | 31 | 62.0 |  | 26 | 86.7 |  | 5 | 25.0 | <0.001 |
|  | >ULN | 19 | 38.0 |  | 4 | 13.3 |  | 15 | 75.0 |  |
|  | ≤ULN ×2 | 46 | 92.0 |  | 29 | 96.7 |  | 17 | 85.0 | 0.289 |
|  | >ULN ×2 | 4 | 8.0 |  | 1 | 3.3 |  | 3 | 15.0 |  |
| Hemoglobin | |  |  |  |  |  |  |  |  |  |
|  | median (range), g/dL | 12.6 (5.8−18) | |  | 13.4 (8.7−15.9) | |  | 11.6 (5.8−18) | | 0.002 |
|  | <12 | 31 | 62.0 |  | 25 | 83.3 |  | 6 | 30.0 | <0.001 |
|  | ≥12 | 19 | 38.0 |  | 5 | 16.7 |  | 14 | 70.0 |  |
| Hypercalcemia | |  |  |  |  |  |  |  |  |  |
|  | median (range), mg/dL | 9.3 (8.6−11.8) | |  | 9.3 (8.6−11.8) | |  | 9.3 (8.8−10.4) | | 0.527 |
|  | No | 44 | 88.0 |  | 27 | 90.0 |  | 17 | 85.0 | 1.000 |
|  | Yes | 2 | 4.0 |  | 1 | 3.3 |  | 1 | 5.0 |  |
|  | Missing | 4 | 8.0 |  | 2 | 6.7 |  | 2 | 10.0 |  |
| Extranodal site, excluding BM | | |  |  |  |  |  |  |  |  |
|  | Negative | 31 | 62.0 |  | 22 | 73.3 |  | 9 | 45.0 | 0.074 |
|  | Positive | 19 | 38.0 |  | 8 | 26.7 |  | 11 | 55.0 |  |
| Bulky disease | |  |  |  |  |  |  |  |  |  |
|  | median (range), cm | 3.7 (1.2−11.6) | |  | 3.7 (1.2−7.8) | |  | 3.7 (1.8−11.6) | | 0.498 |
|  | <6 cm | 38 | 76.0 |  | 25 | 83.3 |  | 13 | 65.0 | 0.289 |
|  | ≥6 cm | 11 | 22.0 |  | 5 | 16.7 |  | 6 | 30.0 |  |
|  | Missing | 1 | 2.0 |  | 0 | 0.0 |  | 1 | 5.0 |  |
| Focal lymph nodal enlargement | |  |  |  |  |  |  |  |  |  |
|  | No^b^ | 39 | .0 |  | 27 | 90.0 |  | 12 | 60.0 | 0.033 |
|  | Yes (≥ 3 cm)^c^ | 3 | 10.0 |  | 3 | 10.0 |  | 7 | 35.0 |  |
|  | No^d^ | 48 | 96.0 |  | 30 | 100.0 |  | 18 | 90.0 | 0.388 |
|  | Yes (≥ 7 cm)^e^ | 1 | 2.0 |  | 0 | 0.0 |  | 1 | 5.0 |  |
|  | Missing | 1 | 2.0 |  | 0 | 0.0 |  | 1 | 5.0 |  |
| SUVmax | |  |  |  |  |  |  |  |  |  |
|  | median (range) | 12.63 (2.61−46.60) | |  | 12.23 (2.61−20.33) | |  | 27.48 (10.41−46.60) | | 0.007 |
|  | Missing | 22 | 44.0 |  | 11 | 36.7 |  | 11 | 55.0 | 0.251 |
|  | FDG-PET/CT | 28 | 56.0 |  | 19 | 63.3 |  | 9 | 45.0 |  |
|  | SUVmax <10 | 7 | 25.0 |  | 7 | 36.8 |  | 0 | 0.0 | 0.062 |
|  | SUVmax ≥10 | 21 | 75.0 |  | 12 | 63.2 |  | 9 | 100.0 |  |
|  | SUVmax <16 | 18 | 64.3 |  | 15 | 78.9 |  | 3 | 33.3 | 0.035 |
|  | SUVmax ≥16 | 10 | 35.7 |  | 4 | 21.1 |  | 6 | 66.7 |  |
|  | SUVmax <20 | 21 | 75.0 |  | 18 | 94.7 |  | 3 | 33.3 | 0.001 |
|  | SUVmax ≥20 | 7 | 25.0 |  | 1 | 5.3 |  | 6 | 66.7 |  |

Abbreviations: ECOG, Eastern Cooperative Oncology Group; LDH, lactate dehydrogenase; ULN, upper limit of normal; BM, bone marrow; SUVmax, maximum standardized uptake value; FDG-PET/CT, ^18^F-fluorodexyglucose positron emission tomography/computed tomography

^a^*P*-value was analyzed by comparing the biopsy-proven FL patients with HT patients.

^b^Not applicable to ^c^, ^c^The nodal mass (≥3 cm) was observed in only one nodal area,

^d^Not applicable to ^e^, ^e^The nodal mass (≥7 cm) was observed in only one nodal area.

Table S2. Distribution of patients stratified by the transformation scoring system in the internal validation cohort

| TSS scores | Non-biopsy patients | | | Cause of death | | |
| --- | --- | --- | --- | --- | --- | --- |
|  | Total | Clinical FL | Clinical transformation | Total | Lymphoma | Others |
|  | (n = 104) | (n = 97) | (n = 7) | (n = 11) | (n = 6) | (n = 5) |
| 0 | 54 | 51 | 3 | 4 | 0 | 4^a^ |
| 1 | 30 | 29 | 1 | 0 | 0 | 0 |
| 2 | 17 | 14 | 3 | 5 | 4 | 1^b^ |
| 3 | 3 | 3 | 0 | 2 | 2 | 0 |
| ≥4 | 0 | 0 | 0 | 0 | 0 | 0 |

Abbreviations: FL, follicular lymphoma; TSS, transformation scoring system.

^a^Unknown (n = 1), lung cancer (n = 1), secondary myelodysplastic syndromes/acute myeloid leukemia (n = 2)

^b^Breast cancer (n = 1)

Table S3. Comparison of the salvage therapy in patients with higher transformation scoring system scores between the derivation cohort and the internal validation cohort

|  |  | Derivation cohort  (n = 28) | |  | Internal validation cohort  (n = 20) | |  |
| --- | --- | --- | --- | --- | --- | --- | --- |
| Numbers | | No. | % |  | No. | % | *P*-value |
| Total number of salvage regimens after disease progression | |  |  |  |  |  |  |
|  | 0 | 0 | 0 |  | 0 | 0 | <0.001 |
|  | 1 | 13 | 46 |  | 9 | 45 |  |
|  | 2 | 11 | 39 |  | 6 | 30 |  |
|  | 3 | 4 | 14 |  | 2 | 10 |  |
|  | 4 | 0 | 0 |  | 3 | 15 |  |
| Rituximab monotherapy | |  |  |  |  |  |  |
|  | 0 | 28 | 100 |  | 14 | 70 | 0.003 |
|  | 1 | 0 | 0 |  | 6 | 30 |  |
| Chemotherapy | |  |  |  |  |  |  |
|  | 0 | 23 | 8*2* |  | 14 | 70 | 0.275 |
|  | 1 | 5 | 18 |  | 4 | 20 |  |
|  | 2 | 0 | 0 |  | 2 | 10 |  |
| R-chemotherapy | |  |  |  |  |  |  |
|  | 0 | 6 | 21 |  | 9 | 45 | 0.140 |
|  | 1 | 20 | 71 |  | 9 | 45 |  |
|  | 2 | 1 | 4 |  | 2 | 10 |  |
|  | 3 | 1 | 4 |  | 0 | 0 |  |
| R- Bendamustine | |  |  |  |  |  |  |
|  | 0 | 24 | 86 |  | 18 | 90 | 1.000 |
|  | 1 | 4 | 14 |  | 2 | 10 |  |
| R- Fludarabine | |  |  |  |  |  |  |
|  | 0 | 28 | 100 |  | 18 | 90 | 0.168 |
|  | 1 | 0 | 0 |  | 2 | 10 |  |
| Radiation | |  |  |  |  |  |  |
|  | 0 | 28 | 100 |  | 17 | 85 | 0.066 |
|  | 1 | 0 | 0 |  | 3 | 15 |  |
| Radiation + chemotherapy | |  |  |  |  |  |  |
|  | 0 | 23 | 82 |  | 18 | 90 | 0.683 |
|  | 1 | 5 | 18 |  | 2 | 10 |  |
| HSCT | |  |  |  |  |  |  |
|  | 0 | 20 | 71 |  | 17 | 85 | 0.319 |
|  | 1 | 8 | 29 |  | 3 | 15 |  |
| Others | |  |  |  |  |  |  |
|  | 0 | 28 | 100 |  | 17 | 85 | 0.066 |
|  | 1 | 0 | 0 |  | 3 | 3 |  |

Abbreviations: R, rituximab; HSCT, hematopoietic stem cell transplantation
