# Supplementary material for: Transformation Scoring System (TSS): A new assessment index for clinical transformation of follicular lymphoma
Source: Cancer Med. 2020 Oct 6;9(23):8864–74. doi: 10.1002/cam4.3501 (PMC7724492; doi:10.1002/cam4.3501)
Supplement: Supplementary file 2 [file CAM4-9-8864-s002.doc]

Table 1. Clinical characteristics of the biopsy-proven follicular lymphoma (FL) or histologic transformation (HT) patients at the time of disease progression in the derivation set

|  |  | Total (N = 76) | |  | FL (n = 42) | |  | HT (n = 34) | |  |
| --- | --- | --- | --- | --- | --- | --- | --- | --- | --- | --- |
| Characteristics | | No. | % |  | No. | % |  | No. | % | *P*-value^a^ |
| Age | |  |  |  |  |  |  |  |  |  |
|  | median (range), years | 61.5 (32−85) | |  | 62.5 (36−85) | |  | 61 (31−83) | | 0.493 |
|  | <61 | 33 | 43.4 |  | 17 | 40.5 |  | 16 | 47.1 | 0.644 |
|  | ≥61 | 43 | 56.6 |  | 25 | 59.5 |  | 18 | 52.9 |  |
| Sex | |  |  |  |  |  |  |  |  |  |
|  | Female | 38 | 50.0 |  | 17 | 40.5 |  | 21 | 61.8 | 0.106 |
|  | Male | 38 | 50.0 |  | 25 | 59.5 |  | 13 | 38.2 |  |
| B symptoms | |  |  |  |  |  |  |  |  |  |
|  | No | 65 | 85.5 |  | 39 | 92.9 |  | 26 | 76.5 | 0.003 |
|  | Yes | 7 | 9.2 |  | 0 | 0.0 |  | 7 | 20.6 |  |
|  | Missing | 4 | 5.3 |  | 3 | 7.1 |  | 1 | 2.9 |  |
| ECOG performance status | |  |  |  |  |  |  |  |  |  |
|  | 0−1 | 65 | 85.5 |  | 40 | 95.2 |  | 25 | 73.5 | 0.001 |
|  | 2−4 | 8 | 10.5 |  | 0 | 0.0 |  | 8 | 23.5 |  |
|  | Missing | 3 | 3.9 |  | 2 | 4.8 |  | 1 | 2.9 |  |
| Ann Arbor Stage | |  |  |  |  |  |  |  |  |  |
|  | I−II | 19 | 25.0 |  | 11 | 26.2 |  | 8 | 23.5 | 0.585 |
|  | III−IV | 45 | 59.2 |  | 21 | 50.0 |  | 24 | 70.6 |  |
|  | Missing | 12 | 15.8 |  | 10 | 23.8 |  | 2 | 5.9 |  |
| LDH | |  |  |  |  |  |  |  |  |  |
|  | median (range), IU/L | 197 (116−5540) | |  | 176.5 (116−288) | |  | 276.5 (143−5540) | | <0.001 |
|  | ≤ULN | 46 | 60.5 |  | 35 | 83.3 |  | 11 | 32.4 | <0.001 |
|  | >ULN | 30 | 39.5 |  | 7 | 16.7 |  | 23 | 67.6 |  |
|  | ≤ULN ×2 | 67 | 88.2 |  | 42 | 100.0 |  | 25 | 73.5 | <0.001 |
|  | >ULN ×2 | 9 | 11.8 |  | 0 | 0.0 |  | 9 | 26.5 |  |
| Hemoglobin | |  |  |  |  |  |  |  |  |  |
|  | median (range), g/dL | 12.85 (4−17.4) | |  | 13.15 (9.8−17.4) | |  | 12.55 (4−15.2) | | 0.011 |
|  | <12 | 20 | 26.3 |  | 6 | 14.3 |  | 14 | 41.2 | 0.010 |
|  | ≥12 | 56 | 73.7 |  | 36 | 85.7 |  | 20 | 58.8 |  |
| White blood cell count | |  |  |  |  |  |  |  |  |  |
|  | median (range), /μL | 5100 (800−40200) | |  | 5100 (2900−40200) | |  | 5050 (800−31000) | | 0.415 |
| Platelet count | |  |  |  |  |  |  |  |  |  |
|  | median (range), ×10^4^ /μL | 17.8 (0.3−51.1) | |  | 18.15 (8.9−39.8) | |  | 17.55 (0.3−51.1) | | 0.758 |
| Hypercalcemia | |  |  |  |  |  |  |  |  |  |
|  | median (range), mg/dL | 9.4 (7.7−12) | |  | 9.4 (8.5−10.3) | |  | 9.4 (7.7−12) | | 0.806 |
|  | No | 74 | 97.4 |  | 42 | 100.0 |  | 32 | 94.1 | 0.197 |
|  | Yes | 2 | 2.6 |  | 0 | 0.0 |  | 2 | 5.9 |  |
| CRP | |  |  |  |  |  |  |  |  |  |
|  | median (range), mg/dL | 0.155 (0.02−26.9) | |  | 0.1 (0.02−4.34) | |  | 0.705 (0.02−26.9) | | 0.001 |
|  | ≤ULN | 36 | 47.4 |  | 25 | 59.5 |  | 11 | 32.4 | 0.022 |
|  | >ULN | 40 | 52.6 |  | 17 | 40.5 |  | 23 | 67.6 |  |
| Bone marrow involvement | |  |  |  |  |  |  |  |  |  |
|  | Negative | 41 | 53.9 |  | 22 | 52.4 |  | 19 | 55.9 | 0.355 |
|  | Positive | 14 | 18.4 |  | 5 | 11.9 |  | 9 | 26.5 |  |
|  | Missing | 21 | 27.6 |  | 15 | 35.7 |  | 6 | 17.6 |  |
| Extranodal site, excluding BM | |  |  |  |  |  |  |  |  |  |
|  | Negative | 50 | 65.8 |  | 31 | 73.8 |  | 19 | 55.9 | 0.080 |
|  | Positive | 24 | 31.6 |  | 9 | 21.4 |  | 15 | 44.1 |  |
|  | Missing | 2 | 2.6 |  | 2 | 4.8 |  | 0 | 0.0 |  |
| Bulky disease | |  |  |  |  |  |  |  |  |  |
|  | median (range), cm | 3.3 (0−12.1) | |  | 2.8 (0−8.2) | |  | 5.6 (0−12.1) | | <0.001 |
|  | <6 cm | 57 | 75.0 |  | 39 | 92.9 |  | 18 | 52.9 | <0.001 |
|  | ≥6 cm | 18 | 23.7 |  | 2 | 4.8 |  | 16 | 47.1 |  |
|  | Missing | 1 | 1.3 |  | 1 | 2.4 |  | 0 | 0.0 |  |
| Focal lymph nodal enlargement | |  |  |  |  |  |  |  |  |  |
|  | No^b^ | 47 | 61.8 |  | 33 | 78.6 |  | 14 | 41.2 | 0.001 |
|  | Yes (≥3 cm)^c^ | 27 | 35.5 |  | 8 | 19.0 |  | 19 | 55.9 |  |
|  | No^d^ | 64 | 84.2 |  | 41 | 97.6 |  | 23 | 67.6 | <0.001 |
|  | Yes (≥7 cm)^e^ | 10 | 13.2 |  | 0 | 0.0 |  | 10 | 29.4 |  |
|  | Missing | 2 | 2.6 |  | 1 | 2.4 |  | 1 | 2.9 |  |
| SUVmax | |  |  |  |  |  |  |  |  |  |
|  | median (range) | 11.63 (2.11−33.34) | |  | 9.20 (2.11−16.7) | |  | 16.74 (4.86−33.34) | | <0.001 |
|  | Missing | 26 | 34.2 |  | 14 | 33.3 |  | 12 | 35.3 | 1.00 |
|  | FDG-PET/CT | 50 | 65.8 |  | 28 | 66.7 |  | 22 | 64.7 |  |
|  | SUVmax <10 | 19 | 38.0 |  | 16 | 57.0 |  | 3 | 13.6 | 0.003 |
|  | SUVmax ≥10 | 31 | 62.0 |  | 12 | 43.0 |  | 19 | 86.4 |  |
|  | SUVmax <16 | 36 | 72.0 |  | 27 | 96.4 |  | 9 | 40.9 | <0.001 |
|  | SUVmax ≥16 | 14 | 28.0 |  | 1 | 3.6 |  | 13 | 59.1 |  |
|  | SUVmax <20 | 45 | 90.0 |  | 28 | 100.0 |  | 17 | 77.3 | 0.012 |
|  | SUVmax ≥20 | 5 | 10.0 |  | 0 | 0.0 |  | 5 | 22.7 |  |
| FLIPI | |  |  |  |  |  |  |  |  |  |
|  | Low risk | 26 | 34.2 |  | 18 | 42.9 |  | 8 | 23.5 | 0.046 |
|  | Intermediate risk | 19 | 25.0 |  | 11 | 26.2 |  | 8 | 23.5 |  |
|  | Poor risk | 28 | 36.8 |  | 10 | 23.8 |  | 18 | 52.9 |  |
|  | Missing | 3 | 3.9 |  | 2 | 4.8 |  | 1 | 2.9 |  |
| IPI | |  |  |  |  |  |  |  |  |  |
|  | Low risk | 27 | 35.5 |  | 19 | 45.2 |  | 8 | 23.5 | <0.001 |
|  | Low-intermediate risk | 27 | 35.5 |  | 18 | 42.9 |  | 9 | 26.5 |  |
|  | High-intermediate risk | 12 | 15.8 |  | 3 | 7.1 |  | 9 | 26.5 |  |
|  | High risk | 7 | 9.2 |  | 0 | 0.0 |  | 7 | 20.6 |  |
|  | Missing | 3 | 3.9 |  | 2 | 4.8 |  | 1 | 2.9 |  |
| Number of relapses from initial diagnosis | | |  |  |  |  |  |  |  |  |
|  | 1 | 53 | 69.7 |  | 33 | 78.6 |  | 20 | 58.8 | 0.081 |
|  | ≥2 | 23 | 30.3 |  | 9 | 21.4 |  | 14 | 41.2 |  |

Abbreviations: ECOG, Eastern Cooperative Oncology Group; LDH, lactate dehydrogenase; ULN, upper limit of normal; CRP, C-reactive protein; BM, bone marrow; SUVmax, maximum standardized uptake value;

FDG-PET/CT, ^18^F-fluorodexyglucose positron emission tomography/computed tomography; FLIPI, Follicular Lymphoma International Prognostic Index; IPI, Internal Prognostic Index

^a^*P*-value was analyzed by comparing the biopsy-proven FL patients with HT patients.

^b^Not applicable to ^c^, ^c^The nodal mass (≥3 cm) was observed in only one nodal area,

^d^Not applicable to ^e^, ^e^The nodal mass (≥7 cm) was observed in only one nodal area.
